# Factors associated with unmet healthcare needs among community-dwelling older adults in South Korea: a cross-sectional study using KNHANES 2023

**DOI:** 10.1186/s12877-026-07330-z

**Published:** 2026-03-11

**Authors:** Heeyoung Hwang, Kyeongmin Jang

**Affiliations:** 1 Myongji St. Mary’s Hospital, Seoul, Republic of Korea; 2 Department of Nursing, College of Health Sciences, Daejin University, 1007 Hoguk-ro, Pocheon-si, Gyeonggi-do 11159 Republic of Korea

**Keywords:** Unmet healthcare needs, Older adults, Andersen’s behavioral model, Health literacy, Health-related quality of life, Functional limitation

## Abstract

**Background:**

Unmet healthcare needs (UHN), defined as perceived need for medical care that is not obtained, are a key indicator of access barriers among older adults. Using recent nationally representative data, this study examined factors associated with UHN among community-dwelling older adults in South Korea within Andersen’s behavioral model framework.

**Methods:**

We conducted a cross-sectional complex-sample analysis of the 2023 Korea National Health and Nutrition Examination Survey (KNHANES; public release December 2024). Adults aged ≥ 65 years with complete data were included (weighted analysis; *n* = 1,393). UHN were assessed by self-report of not receiving needed medical care in the past year (yes/no). Predisposing, enabling, and need-related factors, along with health behaviors, were examined. Hierarchical complex-sample logistic regression models were specified a priori, and adjusted odds ratios (aORs) with 95% confidence intervals (CIs) were estimated.

**Results:**

In the fully adjusted model, lower educational attainment was independently associated with higher odds of UHN (≤ elementary: aOR 3.728, 95% CI 1.31–10.64; middle school: aOR 3.022, 95% CI 1.06–8.61; reference: ≥college). Need-related indicators were also associated with UHN, including dizziness/balance problems (aOR 2.358, 95% CI 1.57–3.53), activity limitation (aOR 1.940, 95% CI 1.23–3.06), and poorer health-related quality of life (per 1-point increase in HINT: aOR 1.071, 95% CI 1.02–1.12). Higher health literacy was inversely associated with UHN (per 1-point increase: aOR 0.912, 95% CI 0.79–0.99). Alcohol use (≥ 1/month) was associated with higher odds of UHN (aOR 3.161, 95% CI 1.95–5.12). Household income, residence, insurance indicators, metabolic syndrome, and current smoking were not statistically significant.

**Conclusions:**

UHN among Korean older adults were associated with educational disadvantage and health-related vulnerability, including mobility limitations and poorer HRQoL. These findings highlight the importance of addressing navigation-related barriers and functional constraints when considering strategies to improve healthcare access in later life.

## Introduction

Unmet healthcare needs (UHN)—situations in which people perceive a need for medical care but do not obtain it—are commonly used as a patient-reported indicator of access barriers and potential inequities in healthcare utilization [1]. UHN is particularly relevant in later life because older adults often require ongoing care for chronic conditions while facing barriers such as mobility constraints, transportation difficulties, and challenges navigating health services [2]. In South Korea, rapid population aging has intensified policy attention to UHN among adults aged 65 years and older, and recent evidence suggests that UHN persists with meaningful variation across sociodemographic and health-related subgroups [3]. Although pandemic-related disruptions highlighted vulnerabilities in healthcare access among older adults, UHN also reflect persistent structural determinants that extend beyond temporary crises [4]. Recent evidence indicates that UHN among older adults are consistently patterned by socioeconomic disadvantage and broader access barriers, including geographic disparities and limitations in local healthcare resources [5]. Social conditions relevant to later life, including social isolation and limited support, have also been linked to unmet medical needs, reinforcing that enduring structural vulnerabilities remain central determinants of access inequities [6].

A widely used framework for organizing determinants of healthcare access is Andersen’s behavioral model, which classifies correlates into predisposing characteristics, enabling resources, and need factors [7]. Enabling resources are central to UHN because they capture the material and structural capacity to obtain care, including economic resources and access-related supports [8]. Residential context may also matter; studies incorporating community-level characteristics have reported geographic disparities in UHN, suggesting that place-based disadvantage can compound individual barriers [6]. In this study, Andersen’s Behavioral Model was used as an explicit analytic framework rather than a descriptive classification scheme. We operationalized (1) predisposing characteristics as accumulated social position and demographic context, (2) enabling resources as structural capacity to obtain care, and (3) need factors as health burden and functional or symptom-related barriers that may impede healthcare use. Accordingly, we specified hierarchical models a priori to evaluate how associations with UHN change as these domains are sequentially incorporated.

Need factors often show the strongest associations with UHN because they represent both care requirements and functional barriers that impede reaching and using services [2]. For example, functional limitations and activity restrictions can complicate appointment-keeping, care coordination, and transportation, and have been associated with higher odds of UHN among older adults [9]. Symptom-specific indicators may be especially informative in older populations; dizziness or balance problems can signal mobility-related constraints and elevated clinical need, potentially increasing the risk of delayed or forgone care [2]. Moreover, health-related quality of life (HRQoL) provides an integrated summary of well-being and vulnerability that may capture broader pathways to UHN; in Korea, HINT-8 has been used and validated as a population-relevant HRQoL measure [10].

Alongside health status and function, health literacy is a plausible correlate of UHN because the ability to obtain, understand, and apply health information can influence navigation, decision-making, and timely care seeking in later life [11]. International evidence suggests that older adults’ unmet healthcare experiences cluster by socioeconomic vulnerability and health burden, emphasizing the importance of jointly considering social position and health-related need [12]. Evidence from Europe also indicates that UHN are linked to perceived health deterioration among older adults, supporting the clinical relevance of UHN as more than a purely administrative access indicator [13].

Despite growing literature, updated evidence from recent Korean national data is still needed to clarify correlates of UHN when socioeconomic position, functional/symptom-related barriers (e.g., dizziness/balance problems), HRQoL (HINT-8), and health literacy are examined simultaneously within a single analytic framework. Prior Korean studies have often relied on earlier survey waves or selected subgroups, and findings regarding enabling resources may differ depending on whether functional constraints and quality-of-life indicators are incorporated [3]. International evidence also suggests that the relative salience of enabling versus need factors may vary across contexts, underscoring the value of re-evaluating determinants using current national data [12]. Therefore, using the 2023 Korea National Health and Nutrition Examination Survey (KNHANES) dataset released in December 2024 and complex-sample analytic procedures, this study examines factors associated with UHN among Korean adults aged ≥ 65 years. Guided by Andersen’s behavioral model, we apply hierarchical adjustment for predisposing factors, enabling resources, and need-related health status—including HRQoL (HINT-8) and health literacy—to provide policy-relevant insights into access barriers in later life.

## Methods

### Study design and data source

This study employed a cross-sectional, complex-sample analysis using data from the 2023 KNHANES. KNHANES is a nationally representative survey conducted annually by the Korea Disease Control and Prevention Agency using a stratified, multistage probability sampling design [14]. The 2023 survey was conducted between January and December 2023, and the dataset was publicly released in December 2024.

KNHANES collects comprehensive information on sociodemographic characteristics, health behaviors, healthcare utilization, health-related quality of life, and clinical measurements through standardized health interviews, examinations, and laboratory tests. Sampling weights, strata, and primary sampling units (PSUs) are provided to enable population-representative inference [15].

### Study population

A total of 6,929 individuals participated in the 2023 KNHANES. Among them, 1,836 participants were aged 65 years or older and were considered eligible for the present study. Participants were excluded if they had missing data on the dependent variable (UHN), key independent variables, or design variables required for complex-sample analysis.

After excluding participants with missing values in variables used for the descriptive and multivariable analyses, 1,393 older adults aged ≥ 65 years constituted the final analytic sample. The analytic flow was as follows: total KNHANES participants → age-eligible participants (≥ 65 years) → complete-case inclusion for key study variables.

### Outcome variable: UHN

UHN were assessed using a self-reported KNHANES questionnaire item asking whether the participant had experienced a situation during the past year in which they needed medical care but did not receive it [16]. Responses were dichotomized as yes or no, with “yes” indicating the presence of UHN. This variable served as the dependent variable in all analyses.

### Independent variables

Independent variables were selected based on the Andersen behavioral model of health service use and grouped into predisposing, enabling, and need-related factors, as well as health behaviors [7]. Enabling factors were limited to economic and insurance-related resources available with sufficient completeness and consistent coding in the public KNHANES 2023 release. Need-related indicators were selected to capture complementary dimensions of health burden (SRH), functional restriction (activity limitation), symptom-related mobility barriers (dizziness/balance problems), and multidimensional HRQoL (HINT-8), while maintaining model parsimony and interpretability.

### Predisposing factors

Predisposing factors included age (years, continuous), sex (male/female), educational attainment, and marital status. Education level was categorized as elementary school or less, middle school, high school, and college or higher. Marital status was categorized as married or widowed/divorced/separated.

### Enabling factors

Enabling factors included household income, area of residence, type of public health insurance, and private health insurance coverage. Household income was categorized into quartiles and analyzed as low, lower-middle, upper-middle, and high income. Area of residence was classified as urban (dong) or rural (eup/myeon). Public health insurance type was categorized as National Health Insurance or Medical Aid. Private health insurance coverage was classified as yes or no.

### Need-related factors (health status)

Need-related factors included self-rated health status, balance or dizziness problems, activity limitation, metabolic syndrome, and health-related quality of life. Balance or dizziness problems were assessed by self-report of symptoms during the past year (yes/no). Activity limitation was defined as self-reported limitation in daily or social activities due to health problems (yes/no). Self-rated health was categorized as very good, good, fair, poor, or very poor.

Health-related quality of life was measured using the Health-related Quality of Life Instrument with 8 Items (HINT). The HINT score was treated as a continuous variable, with higher scores indicating poorer health-related quality of life [10].

Metabolic syndrome was defined according to standard KNHANES criteria based on measured waist circumference, blood pressure, fasting glucose, triglycerides, and high-density lipoprotein cholesterol [17]. Health literacy was assessed using the KNHANES health literacy measure (total score range: [4–40]), with higher scores indicating higher health literacy. The score was treated as a continuous variable and entered per 1-point increase in the regression models. Participants with missing values on the health literacy score were excluded from the complete-case analytic sample.

### Health behaviors

Health behaviors included alcohol use and smoking status. Alcohol use was defined as consuming alcohol at least once per month during the past year (yes/no). Smoking status was categorized as current smoker or non-current smoker (former or never smoker).

### Statistical analysis

All analyses accounted for the complex sampling design of the KNHANES by incorporating sampling strata, primary sampling units (PSUs), and sampling weights. Descriptive statistics were used to summarize participant characteristics, and group differences according to UHN were examined using complex-sample chi-square tests for categorical variables and complex-sample general linear models for continuous variables.

Complex-sample logistic regression analyses were conducted using theory-driven hierarchical models. Model 1 included predisposing factors; Model 2 additionally incorporated enabling factors; and Model 3 further included need-related factors, health behaviors, health-related quality of life (HINT), and health literacy. All models were specified a priori according to Andersen’s Behavioral Model, and no automated stepwise variable selection procedure was applied.

To assess potential multicollinearity among health-related variables included in Model 3, variance inflation factors (VIFs) were calculated. All VIF values ranged from 0.9 to 1.4, indicating no evidence of problematic multicollinearity.

Adjusted odds ratios (aORs) with 95% confidence intervals (CIs) were estimated. Model fit was evaluated using pseudo R² statistics (Cox & Snell, Nagelkerke, and McFadden). All analyses were performed using SPSS Statistics for Windows, version 30.0 (IBM Corp., Armonk, NY, USA), with statistical significance set at *p* < .05.

### Ethical considerations

The Korea National Health and Nutrition Examination Survey (KNHANES) was conducted in accordance with the Declaration of Helsinki and approved by the Institutional Review Board of the Korea Disease Control and Prevention Agency (KDCA IRB No. 2022-11-16-R-A; approved November 16, 2022). All participants provided written informed consent prior to participation. The present study involved secondary analysis of publicly available, fully de-identified data from the 9th KNHANES (2022–2023). In addition, the study protocol was reviewed by the Institutional Review Board of Daejin University and was granted an exemption from ethics review (IRB No. 1040656-202512-HR-02-18; approved December 2, 2025).

## Results

### Characteristics of older adults according to UHN

Table 1 summarizes the weighted characteristics of Korean older adults (≥ 65 years) by UHN status. Age did not differ between groups (mean 72.61 [SE 0.51] in the no-UHN group vs. 72.34 [SE 0.54] in the UHN group; *p* = .621), and no significant differences were observed for sex (*p* = .082) or marital status (*p* = .334). Educational attainment differed markedly by UHN (*p* < .001); the UHN group had a higher proportion with elementary education or less (61.6% vs. 42.5%) and a lower proportion with college education or higher (5.2% vs. 13.2%).

**Table 1:** Weighted characteristics of older adults aged ≥ 65 years by unmet healthcare needs status (*N* = 1,393)

Variable	Category / Unit	Total	No unmet HC	Unmet HC	*p*
Predisposing					
Sex	Male	44.3	45.1	37.5	0.082
	Female	55.7	54.9	62.5	
Education	≤ Elementary	44.5	42.5	61.6	< 0.001
	Middle school	20.6	20.4	21.8	
	High school	22.6	23.9	11.4	
	≥ College	12.4	13.2	5.2	
Marital status	Married	69.9	70.3	66.2	0.334
	Widowed/Divorced	30.1	29.7	33.8	
Age (years)	Mean ± SE	72.37 ± 0.51	72.61 ± 0.51	72.34 ± 0.54	0.621
Enabling					
Household income	Low	42.4	41.4	49.4	0.029
	Lower-middle	29.4	29	31.8	
	Upper-middle	18.4	18.8	14.1	
	High	9.9	10.7	4.7	
Area of residence	Urban	76.7	76.8	75.7	0.799
	Rural	23.3	23.2	24.3	
Insurance type	NHI	94	94	91.4	0.171
	Medical Aid	6	6	8.6	
Private insurance	Yes	62.7	63.7	54.2	0.040
	No	37.3	36.3	45.8	
Need (health)					
Balance/dizziness	Yes	41.9	38.9	67	< 0.001
	No	58.1	61.1	33	
Activity limitation	Yes	14.7	12.8	31.6	< 0.001
	No	85.3	87.2	68.4	
Self-rated health	Very good	6.5	6.7	4.3	< 0.001
	Good	20.8	21.8	12.8	
	Fair	47.1	48.1	38.6	
	Poor	17.3	16.7	22.2	
	Very poor	8.3	6.7	22.2	
Metabolic syndrome	No	72.6	72.7	71.6	0.813
	Yes	27.4	27.3	28.4	
HINT	Mean ± SE	15.09 ± 0.41	14.81 ± 0.42	17.50 ± 0.41	< 0.001
Health literacy score	Mean ± SE	27.65 ± 0.20	27.77 ± 0.23	25.75 ± 0.21	0.044
Behaviors					
Alcohol use	Yes	51.9	29.1	53.3	< 0.001
	No	48.1	70.9	46.7	
Current smoking	Current	10.5	10.1	14.1	0.188
	Non-current	89.5	89.9	85.9	

Values are weighted % or mean (SE) accounting for the complex sampling design; Rao–Scott χ² tests were used for categorical variables and complex-sample GLM for continuous variables

*Abbreviations*: *HC* Healthcare, *HRQoL* Health-related quality of life, *HINT* Health-related quality of life index, *NHI* National Health Insurance, *SE* Standard error

Among enabling factors, household income distribution differed by UHN (*p* = .029), with a higher proportion in the lowest income quartile in the UHN group (49.4% vs. 41.4%) and a lower proportion in the highest quartile (4.7% vs. 10.7%). Private health insurance coverage was less common among participants with UHN (54.2% vs. 63.7%; *p* = .040), whereas area of residence (*p* = .799) and public insurance type (*p* = .171) were not associated with UHN.

Regarding need-related factors, balance/dizziness problems (67.0% vs. 38.9%; *p* < .001) and activity limitation (31.6% vs. 12.8%; *p* < .001) were more prevalent in the UHN group. Self-rated health also differed significantly across groups (*p* < .001), with “very poor” health reported more frequently among those with UHN (22.2% vs. 6.7%). Metabolic syndrome prevalence did not differ by UHN status (*p* = .813). Participants with UHN had poorer HRQoL as indicated by higher HINT scores (17.50 [SE 0.41] vs. 14.81 [SE 0.42]; *p* < .001) and lower health literacy scores (25.75 [SE 0.21] vs. 27.77 [SE 0.23]; *p* = .044). Alcohol use differed between groups (*p* < .001), while current smoking did not (*p* = .188).

### Stepwise associations between predisposing, enabling, and need factors and UHN

Table 2 shows stepwise complex-sample logistic regression models for UHN. In Model 1, education was associated with UHN: compared with ≥college, ≤elementary (aOR 3.731, 95% CI 1.416–9.804) and middle school (aOR 2.681, 95% CI 1.043–6.897) showed higher odds. Other predisposing variables were not significant (Table 2).

**Table 2:** Hierarchical complex-sample logistic regression models for unmet healthcare needs among adults aged ≥ 65 years (*N* = 1,393)

Predictor	Model 1 aOR (95% CI)	Model 2 aOR (95% CI)	Model 3 aOR (95% CI)
Predisposing factors			
Age (per 1-year increase)	0.989 (0.950–1.030)	0.967 (0.922–1.015)	0.963 (0.910–1.020)
Male (vs. Female)	0.921 (0.629–1.350)	0.935 (0.646–1.350)	0.773 (0.502–1.192)
Education: ≤Elementary (vs. ≥College)	3.731 (1.416–9.804)	4.425 (1.587–12.346)	3.731 (1.307–10.638)
Education: Middle school (vs. ≥College)	2.681 (1.043–6.897)	3.040 (1.080–8.547)	3.021 (1.062–8.621)
Education: High school (vs. ≥College)	1.206 (0.451–3.226)	1.447 (0.510–4.115)	1.412 (0.484–4.115)
Married (vs. Widowed/Divorced)	1.025 (0.678–1.548)	1.117 (0.736–1.697)	1.062 (0.657–1.712)
Enabling factors			
Income: Low (vs. High)	—	2.404 (0.980–5.882)	1.942 (0.804–4.673)
Income: Lower-middle (vs. High)	—	2.519 (1.068–5.917)	2.119 (0.894–5.025)
Income: Upper-middle (vs. High)	—	2.294 (0.870–6.024)	1.887 (0.742–4.808)
Area: Urban (vs. Rural)	—	1.144 (0.700–1.869)	1.129 (0.685–1.858)
National Health Insurance (vs. Medical Aid)	—	1.507 (0.433–1.897)	1.799 (0.766–4.219)
Private health insurance: Yes (vs. No)	—	0.713 (0.449–1.134)	0.659 (0.383–1.134)
Need factors / health behaviors			
Dizziness/balance problem: Yes (vs. No)	—	—	2.358 (1.575–3.534)
Activity limitation: Yes (vs. No)	—	—	1.938 (1.227–3.067)
Self-rated health: Very good (vs. Very poor)	—	—	0.792 (0.251–2.493)
Self-rated health: Good (vs. Very poor)	—	—	0.487 (0.224–1.058)
Self-rated health: Fair (vs. Very poor)	—	—	0.476 (0.274–0.825)
Self-rated health: Poor (vs. Very poor)	—	—	0.472 (0.250–0.893)
Metabolic syndrome: No (vs. Yes)	—	—	1.149 (0.668–1.976)
Alcohol drinking (≥ 1/month): Yes (vs. No)	—	—	3.165 (1.949–5.128)
Current smoking: Current (vs. Non)	—	—	1.464 (0.758–2.825)
HINT (per 1-point increase)	—	—	1.071 (1.020–1.124)
Health literacy score (per 1-point increase)	—	—	0.912 (0.788–0.986)

Complex-sample logistic regression accounted for sampling strata, primary sampling units, and weights. Adjusted odds ratios (aORs) with 95% confidence intervals (CIs) are presented for Models 1–3. Model fit improved across steps (Model 1: Wald F = 2.31, *p* = .018; Nagelkerke R² = 0.044; Model 2: Wald F = 2.74, *p* = .003; Nagelkerke R² = 0.062; Model 3: Wald F = 5.85, *p* < .001; Nagelkerke R² = 0.209)

*Abbreviations*: *aOR*  Adjusted odds ratio, *CI*  Confidence interval, *HINT*  Health-related quality of life index

In Model 2, enabling factors were added. Compared with the highest income group, lower-middle income was associated with higher odds (aOR 2.519, 95% CI 1.068–5.917), whereas low and upper-middle income were not significant. Education effects for ≤elementary (aOR 4.425, 95% CI 1.587–12.346) and middle school (aOR 3.040, 95% CI 1.080–8.547) remained significant (Table 2).

In Model 3, need factors and health behaviors were added. Balance/dizziness problems (aOR 2.358, 95% CI 1.575–3.534), activity limitation (aOR 1.938, 95% CI 1.227–3.067), alcohol drinking ≥ 1/month (aOR 3.165, 95% CI 1.949–5.128), higher HINT score (per 1-point increase: aOR 1.071, 95% CI 1.020–1.124), and health literacy score (per 1-point increase: aOR 0.912, 95% CI 0.788–0.986) were associated with UHN. Compared with very poor self-rated health, fair (aOR 0.476, 95% CI 0.274–0.825) and poor (aOR 0.472, 95% CI 0.250–0.893) self-rated health were associated with lower odds (Table 2). Model fit indices improved across the three models (Table 2).

### Factors independently associated with UHN in the fully adjusted model

Table 3 summarizes the fully adjusted model. After adjusting for all covariates, education remained significantly associated with UHN: ≤elementary (aOR 3.728, 95% CI 1.31–10.64; *p* = .013) and middle school (aOR 3.022, 95% CI 1.06–8.61; *p* = .037) were associated with higher odds compared with ≥college.

**Table 3:** Complex-sample logistic regression predicting unmet healthcare needs among adults aged ≥ 65 years (*N* = 1,393)

Variable	B	SE	Wald χ²	*p*	aOR	95% CI
Predisposing factors						
Age (per 1-year)	−0.037	0.029	1.628	0.202	0.964	0.91–1.02
Male (ref: Female)	−0.257	0.219	1.377	0.241	0.773	0.50–1.19
Elementary or less (ref: ≥College)	1.316	0.531	6.142	0.013	3.728	1.31–10.64
Middle school (ref: ≥College)	1.106	0.53	4.355	0.037	3.022	1.06–8.61
High school (ref: ≥College)	0.345	0.542	0.404	0.525	1.411	0.48–4.12
Married (ref: Widowed/Divorced)	0.06	0.242	0.06	0.806	1.061	0.66–1.71
Enabling factors						
Income (Low; ref: High)	0.663	0.446	2.21	0.137	1.941	0.80–4.68
Income (Lower-middle; ref: High)	0.751	0.437	2.953	0.086	2.119	0.89–5.02
Income (Upper-middle; ref: High)	0.635	0.473	1.802	0.179	1.887	0.74–4.80
Urban (ref; Rural)	0.121	0.253	0.229	0.632	1.129	0.68–1.86
NHI (ref: Medical Aid)	0.587	0.432	1.846	0.174	1.799	0.77–4.22
Private insurance: Yes (ref: No)	−0.417	0.274	2.316	0.128	0.659	0.38–1.13
Need factors						
Balance/dizziness: Yes (ref: No)	0.858	0.204	17.689	< 0.001	2.358	1.57–3.53
Activity limitation: Yes (ref: No)	0.663	0.232	8.154	0.004	1.94	1.23–3.06
SRH: Very good (ref: Very poor)	−0.234	0.581	0.162	0.688	0.792	0.25–2.50
SRH: Good (ref: Very poor)	−0.719	0.392	3.364	0.067	0.487	0.22–1.06
SRH: Fair (ref: Very poor)	−0.744	0.279	7.102	0.008	0.475	0.27–0.82
SRH: Poor (ref: Very poor)	−0.750	0.322	5.425	0.020	0.472	0.25–0.89
MetS: No (ref: Yes)	0.14	0.275	0.257	0.612	1.15	0.67–1.98
HINT (per 1-point)	0.069	0.025	7.508	0.006	1.071	1.02–1.12
Health literacy score (per 1-point)	−0.092	0.031	6.752	0.041	0.912	0.79–0.99
Health behaviors						
Alcohol use: Yes (ref: No)	1.151	0.245	22.071	< 0.001	3.161	1.95–5.12
Current smoking (ref: Non)	0.382	0.333	1.313	0.252	1.464	0.76–2.83

Complex-sample logistic regression (outcome: unmet healthcare needs in the past year, Yes); analyses accounted for sampling strata, PSUs, and weights. Model fit: Wald F (modified) = 5.850, df = (23, 132), *p* < .001; pseudo R² = 0.102 (Cox & Snell), 0.209 (Nagelkerke), 0.161 (McFadden); unweighted *n* = 1,393

*Abbreviations*: *aOR*  Adjusted odds ratio, *CI*  Confidence interval, *HINT*  Health-related quality of life index, *MetS*  Metabolic syndrome, *NHI*  National Health Insurance, *PSU*  Primary sampling unit, *SE*  Standard error, *SRH*  Self-rated health

Among need-related factors, balance/dizziness problems (aOR 2.358, 95% CI 1.57–3.53; *p* < .001), activity limitation (aOR 1.94, 95% CI 1.23–3.06; *p* = .004), higher HINT score (per 1-point: aOR 1.071, 95% CI 1.02–1.12; *p* = .006), and health literacy score (per 1-point: aOR 0.912, 95% CI 0.79–0.99; *p* = .041) were associated with UHN. Compared with very poor self-rated health, fair (aOR 0.475, 95% CI 0.27–0.82; *p* = .008) and poor (aOR 0.472, 95% CI 0.25–0.89; *p* = .020) were associated with lower odds. Among health behaviors, alcohol use was associated with UHN (aOR 3.161, 95% CI 1.95–5.12; *p* < .001), whereas current smoking was not significant (*p* = .252). Age, sex, marital status, household income categories, urban residence, insurance type, private insurance, and metabolic syndrome were not significant in the fully adjusted model (Table 3).

## Discussion

Using nationally representative KNHANES 2023 data and theory-driven hierarchical logistic regression grounded in Andersen’s behavioral model, this study identified independent correlates of unmet healthcare needs (UHN) among Korean adults aged ≥ 65 years. In the fully adjusted model, lower educational attainment and several need-related indicators—particularly dizziness/balance problems, activity limitation, and poorer HRQoL (higher HINT score)—were associated with higher odds of UHN. Lower health literacy and monthly alcohol use were also associated with UHN, whereas income, residential area, insurance indicators, metabolic syndrome, and smoking were not statistically significant.

Although HINT, self-rated health, and activity limitation are conceptually related, they represent complementary dimensions of global perceived health, functional restriction, and multidimensional quality of life. Multicollinearity diagnostics (VIF range: 0.9–1.4) did not indicate instability, supporting interpretation of HRQoL as an independent correlate. While alternative model specifications excluding HINT or individual health indicators could be considered, our objective was to examine independent associations within a unified Andersen-model framework, and the stability of estimates supports this specification.

### Educational inequality as a persistent predisposing determinant of UHN

Educational attainment showed a persistent, independent relationship with UHN in the fully adjusted model. This pattern is consistent with Korean evidence indicating that older adults with lower socioeconomic position—particularly lower education—experience higher unmet medical need. For example, Roh and Kim (2023) reported differences in prevalence and reasons for UHN among older Koreans before and during the early COVID-19 period, emphasizing sociodemographic disparities in unmet care experiences [1]. Likewise, Eo and Um (2024) analyzed older adults and found that multiple factors—including sociodemographic vulnerabilities—shape UHN, reinforcing the relevance of education beyond material resources alone [3].

Education may operate through mechanisms aligned with Andersen’s “predisposing characteristics,” but its effect likely reflects downstream pathways such as communication efficacy, system navigation capacity, and the ability to act on health information when barriers arise. During the COVID-19 era, international studies similarly suggested that UHN clusters by vulnerability and health burden, implying that predisposing disadvantages can amplify the impact of situational disruptions on access [12]. In this context, the persistence of educational gradients in our fully adjusted model suggests that educational inequality is not merely a proxy for current income or insurance status but may capture cumulative disadvantage that constrains older adults’ ability to convert perceived need into realized care.

### Enabling resources: why income effects may attenuate after accounting for need and function

In Model 2, lower household income levels were associated with higher odds of UHN, but these associations weakened after adding need-related factors and continuous health measures. This attenuation is plausible in older populations because functional status and symptom burden can simultaneously increase perceived need while also creating practical obstacles to obtaining care. In other words, the observed “income gradient” in unmet need may partially reflect differential distribution of health burden and functional limitations across income strata, rather than purely affordability barriers.

Korean and international literature supports the idea that enabling resources and need factors interact and that the relative importance of enabling versus need may shift depending on whether functional constraints are included. Eo and Um noted the importance of considering multiple determinants together to understand unmet needs among older adults [3]. Beyond Korea, a large systematic review and meta-analysis of unmet needs among older adults highlighted that unmet need is multi-determined and that functional and health-related factors frequently show strong associations across settings [18]. Taken together, our stepwise pattern reinforces the practical value of hierarchical modeling aligned with Andersen’s framework: enabling resources matter, but their apparent effects can be substantially reduced when symptom/functional barriers and HRQoL are simultaneously modeled.

### Need-related barriers: dizziness/balance problems and activity limitation as high-impact correlates

Dizziness/balance problems and activity limitation were strongly associated with UHN in the fully adjusted model. Dizziness is common among older adults and is linked to vulnerability profiles (e.g., ADL and HRQoL differences) in populations at risk [19]. Dizziness has also been associated with subsequent falls and fall-related outcomes, supporting its clinical relevance as a marker of mobility risk [20]. Moreover, older adults may receive less treatment for vertigo/dizziness than younger adults, which could contribute to delayed or suboptimal management [21].

Activity limitation is a direct functional barrier to accessing services and may reflect disability, multimorbidity, or frailty. A population-based KNHANES analysis reported that perceived activity restriction was associated with higher odds of unmet medical needs among middle-aged and older adults, supporting a functional-limitation pathway to unmet care [22]. These findings collectively point to the value of mobility-sensitive access supports (e.g., transportation assistance, outreach, simplified scheduling) for older adults with dizziness/balance symptoms or activity limitations.

### Health-related quality of life (HINT-8) as an integrative marker of vulnerability to UHN

Higher HINT-8 scores (poorer HRQoL) were associated with increased odds of UHN, suggesting that unmet need is not only a function of discrete symptoms or diagnoses but also an integrative experience of diminished well-being and functioning. Importantly, HINT-8 has been validated for older Korean populations, supporting its use as a population-relevant HRQoL measure in national surveys and older-adult research [23]. HRQoL may capture cumulative effects of multimorbidity, psychological distress, pain, fatigue, and social constraints, all of which can heighten perceived need while simultaneously making healthcare utilization more difficult.

International evidence further supports the clinical importance of UHN as more than an “administrative” access measure. Smolić et al. showed that UHN during the COVID-19 period were associated with subsequent worsening of self-reported health, indicating that unmet need can translate into perceived health deterioration among older adults [13]. Moreover, typology-based European work suggests that unmet need experiences cluster in meaningful patterns, often aligning with health burden and vulnerability profiles [12]. In this context, the HINT-8 association in our study strengthens the argument that UHN reflects a clinically meaningful “access-vulnerability” phenotype among older adults, potentially suitable for screening and targeted support.

### Residential context and place-based disadvantage: interpretation of non-significant area effects

In descriptive comparisons, area of residence did not significantly differ by UHN status, and residence was not highlighted as an independent correlate in the fully adjusted model. This does not necessarily imply that geography is irrelevant; rather, it may indicate that individual-level enabling and need-related variables (e.g., symptoms, activity limitation, HRQoL) account for much of the variability that might otherwise be attributed to urban–rural residence in this dataset. Nonetheless, place-based disadvantage remains an important concern, especially when community-level infrastructure and demographic decline may shape access.

A multilevel and spatial analysis in Korea integrating a community “local extinction” index found that unmet healthcare experiences are associated with both individual and community-level factors, suggesting that geographic vulnerability can compound access barriers beyond individual characteristics [6]. Because KNHANES is optimized for national representativeness rather than detailed local context, future studies could link older adults’ UHN to richer community-level indicators (e.g., facility density, travel time, transport networks) to better capture place-based mechanisms.

### Health literacy and health behaviors: implications for navigation-sensitive interventions

Although the study design incorporated health literacy in the hierarchical framework, the central fully adjusted findings emphasized education, dizziness/balance problems, activity limitation, HRQoL, and alcohol use. Health literacy remains conceptually relevant because it affects navigation, timely decision-making, and understanding of care pathways in later life. Evidence from older adults shows that health literacy is meaningfully tied to concerns about unmet medical needs and related well-being pathways [11]. Even when not statistically dominant in a given multivariable model, health literacy can be an actionable lever for intervention—particularly through simplified communication, patient navigation programs, and tailored outreach for older adults with limited educational background.

Alcohol use was also associated with UHN in the fully adjusted model. One plausible interpretation is that alcohol use may act as a marker of broader lifestyle and psychosocial vulnerability (e.g., sleep disturbance, medication interactions, fall risk, or social context), which may complicate timely care seeking. While direct evidence connecting alcohol use to unmet medical care in older adults is less consistent across settings, the finding highlights the need to consider behavioral risk and care engagement together, especially when combined with dizziness/balance symptoms that may be exacerbated by alcohol-related physiologic changes in older age.

### Policy and practice implications

Collectively, these findings suggest that policy responses to UHN among older Korean adults are patterned by educational disadvantage and mobility-related health vulnerability. First, the persistent educational gradient indicates that universal insurance coverage alone may be insufficient to eliminate inequities in realized access; navigation assistance and simplified communication strategies may be particularly relevant for older adults with lower educational attainment. Second, the associations observed for dizziness/balance problems and activity limitation highlight the potential importance of mobility-sensitive access supports, such as transportation assistance, community outreach, and appointment systems adapted to functional constraints. Third, the independent association of HRQoL (HINT-8) with UHN suggests that integrative vulnerability markers may help identify older adults at elevated risk of unmet need, even when traditional enabling indicators are not strongly predictive.

### Strengths and limitations

This study has several strengths, including the use of the most recent nationally representative data (KNHANES 2023), application of complex-sample analytic procedures accounting for survey design, and a theory-driven hierarchical modeling strategy grounded in Andersen’s Behavioral Model. However, several limitations warrant consideration. First, the cross-sectional design precludes causal inference, and the directionality between UHN and health-related quality of life (HRQoL) cannot be established. Second, UHN was self-reported and may be affected by recall bias or reporting heterogeneity, potentially varying by education or health status. Third, although we incorporated multiple domains consistent with Andersen’s framework, several relevant enabling factors (e.g., transportation access, living arrangements, and social support) were not comprehensively captured in the available KNHANES variables. In addition, we did not include a full range of disease-specific diagnoses (e.g., cardiovascular, mental health, or musculoskeletal disorders) to maintain parsimony and limit overadjustment, and residual confounding cannot be excluded. Finally, the complete-case approach may introduce selection bias, and KNHANES provides limited contextual information on local healthcare infrastructure, which may underestimate community-level access barriers. Future longitudinal and multilevel studies are needed to clarify temporal ordering and contextual mechanisms underlying unmet healthcare needs.

## Conclusion

In conclusion, using KNHANES 2023 data, this study demonstrated that UHN among Korean older adults was associated with lower education and with need-related barriers—particularly dizziness/balance problems, activity limitation, and poorer HRQoL measured by HINT-8—within a hierarchical Andersen-model framework. These findings indicate that UHN in later life cluster with health-related vulnerability and reduced navigation capacity, beyond financial access alone. Although causal inference is not possible given the cross-sectional design, the results may inform efforts to identify older adults at higher risk of unmet care and guide discussions on improving access support in aging populations.

## Data Availability

The dataset supporting the conclusions of this article is publicly available from the Korea National Health and Nutrition Examination Survey (KNHANES) repository, Korea Disease Control and Prevention Agency. https://knhanes.kdca.go.kr.

## References

[1] Roh HL, Kim SD. Unmet Healthcare Needs among the Elderly Korean Population: Before and during the COVID-19 Pandemic. Syst vol. 2023;11:437.

[2] Kim S, Hwang J. What are the factors affecting older adults’ experience of unmet healthcare needs amid the COVID-19 pandemic in Korea? BMC Geriatr. 2023;23(1):517. 37626287 10.1186/s12877-023-04208-2 PMC10463954

[3] Eo YS, Um J. Unmet healthcare needs among older people in South Korea. International Journal of Healthcare Management. 2026;19(1):1–10. 10.1080/20479700.2024.2396768.

[4] Hwang J, Kim S. How do perceptions of public health measures affect experience of unmet healthcare needs among older Korean adults during COVID-19 pandemic? Prev Med Rep. 2022;26:101735. 35198363 10.1016/j.pmedr.2022.101735 PMC8850269

[5] Kwon Y, Sohn M, Choi M. Unmet healthcare needs and the local extinction index: an analysis of regional disparities impacting South Korea’s older adults. Frontiers in Public Health. 2024;12:1423108. 10.3389/fpubh.2024.1423108. 10.3389/fpubh.2024.1423108 PMC11325592 39148647

[6] Yang JM, Lee SK, Kim JH. Association between objective social isolation and unmet medical needs: a nationwide cross-sectional study in Korea. J Prev Med Public Health. 2024;57(3):242–51. 10.3961/jpmph.23.516. 38697912 10.3961/jpmph.23.516 PMC11164600

[7] Park M-J, Chung M-Y. Factors affecting unmet healthcare needs in female baby boomers: Andersen model application in Korea. PLoS ONE. 2023;18(6):e0286425. 37262054 10.1371/journal.pone.0286425 PMC10234522

[8] Quintal C, Moura Ramos L, Antunes M, Lourenço Ó. Unmet healthcare needs among the population aged 50 + and their association with health outcomes during the COVID-19 pandemic. Eur J Ageing. 2023;20(1):12. 37119316 10.1007/s10433-023-00758-x PMC10148617

[9] Hyejin L, Bumjo O, Sunyoung K, Kiheon L. ADL/ IADL dependencies and unmet healthcare needs in older persons: A nationwide survey. Arch Gerontol Geriatr. 2021;96:104458. 34147824 10.1016/j.archger.2021.104458

[10] Kim J, Jo MW, Lee HJ, Ahn SH, Son BH, Lee JW, Lee SB. Validity and reliability of the Health-Related Quality of Life Instrument with 8 Items (HINT-8) in Korean breast cancer patients. Osong Public Health Res Perspect. 2021;12(4):254–63. 34465074 10.24171/j.phrp.2021.0005 PMC8408420

[11] Li J, Wang Q, Zhou X. Health literacy, worry about unmet needs for medical care, and psychological well-being among older Chinese adults. Geriatr Gerontol Int. 2024;24(Suppl 1):202–7. 38050461 10.1111/ggi.14754 PMC11503555

[12] Smolić Š, Međimurec P, Čipin I, Mudražija S, Mustač D, Fabijančić M. The hidden crisis: classifying unmet healthcare needs in European older adults during COVID-19. Eur J Ageing. 2025;22(1):31. 40601053 10.1007/s10433-025-00866-w PMC12222597

[13] Smolić Š, Blaževski N, Fabijančić M. The Impact of Unmet Healthcare Needs on the Perceived Health Status of Older Europeans During COVID-19. Int J Public Health. 2024;69:1607336. 39403568 10.3389/ijph.2024.1607336 PMC11471687

[14] Oh K, Kim Y, Kweon S, Kim S, Yun S, Park S, Lee YK, Kim Y, Park O, Jeong EK. Korea National Health and Nutrition Examination Survey, 20th anniversary: accomplishments and future directions. Epidemiol Health. 2021;43:e2021025. 33872484 10.4178/epih.e2021025 PMC8289475

[15] Kweon S, Kim Y, Jang M-j, Kim Y, Kim K, Choi S, Chun C, Khang Y-H, Oh K. Data Resource Profile: The Korea National Health and Nutrition Examination Survey (KNHANES). Int J Epidemiol. 2014;43(1):69–77. 24585853 10.1093/ije/dyt228 PMC3937975

[16] Jung B, Ha IH. Determining the reasons for unmet healthcare needs in South Korea: a secondary data analysis. Health Qual Life Outcomes. 2021;19(1):99. 33743725 10.1186/s12955-021-01737-5 PMC7981839

[17] Seo MH, Lee WY, Kim SS, Kang JH, Kang JH, Kim KK, Kim BY, Kim YH, Kim WJ, Kim EM, et al. 2018 Korean Society for the Study of Obesity Guideline for the Management of Obesity in Korea. J Obes Metab Syndr. 2019;28(1):40–5. 31089578 10.7570/jomes.2019.28.1.40 PMC6484940

[18] Rahman MM, Rosenberg M, Flores G, Parsell N, Akter S, Alam MA, Rahman MM, Edejer T. A systematic review and meta-analysis of unmet needs for healthcare and long-term care among older people. Health Econ Rev. 2022;12(1):60. 36482044 10.1186/s13561-022-00398-4 PMC9733388

[19] Kammerlind A-S, Peolsson A, Johansson MM. Dizziness in older persons at high risk of future hospitalization: prevalence, differences between those with and without dizziness, and effect of a proactive primary care intervention. BMC Geriatr. 2022;22(1):315. 35399055 10.1186/s12877-022-02910-1 PMC8996541

[20] Li Y, Smith RM, Whitney SL, Seemungal BM, Ellmers TJ. Association between dizziness and future falls and fall-related injuries in older adults: a systematic review and meta-analysis. Age Ageing. 2024;53(9):afae177. 39293812 10.1093/ageing/afae177 PMC11410394

[21] Prell T, Finn S, Axer H. How healthcare utilization due to dizziness and vertigo differs between older and younger adults. Front Med. 2022;9:852187. 10.3389/fmed.2022.852187 PMC8889010 35252281

[22] Yang J-M, Kim M-S, Hong J-S, Kim J-H. Association between Perceived Activity Restriction Due to People’s Perception of Aging and Unmet Medical Needs among Middle-Aged and Elderly People: A Population-Based Study. Med vol. 2024;60:87. 10.3390/medicina60010087 PMC10818869 38256348

[23] Kim S-H, Kim M. Validity of the health-related quality of life instrument with 8 items (HINT-8) in the Korean elderly: a cross-sectional study. J Korean Gerontological Nurs. 2022;24(3):248–56.

